# Prion Protein in Milk

**DOI:** 10.1371/journal.pone.0000071

**Published:** 2006-12-20

**Authors:** Nicola Franscini, Ahmed El Gedaily, Ulrich Matthey, Susanne Franitza, Man-Sun Sy, Alexander Bürkle, Martin Groschup, Ueli Braun, Ralph Zahn

**Affiliations:** 1 Alicon AG Schlieren, Switzerland; 2 Institute of Pathology, Biomedical Research Building, Case Western University School of Medicine Cleveland, Ohio, United States of America; 3 Lehrstuhl Molekulare Toxikologie, University of Konstanz Konstanz, Germany; 4 Friedrich-Loeffler-Institut, Bundesforschungsinstitut für Tiergesundheit Greifswald, Gemany; 5 Departement für Nutztiere, University of Zurich Zurich, Switzerland; University of Liverpool, United Kingdom

## Abstract

**Background:**

Prions are known to cause transmissible spongiform encephalopathies (TSE) after accumulation in the central nervous system. There is increasing evidence that prions are also present in body fluids and that prion infection by blood transmission is possible. The low concentration of the proteinaceous agent in body fluids and its long incubation time complicate epidemiologic analysis and estimation of spreading and thus the risk of human infection. This situation is particularly unsatisfactory for food and pharmaceutical industries, given the lack of sensitive tools for monitoring the infectious agent.

**Methodology/Principal Findings:**

We have developed an adsorption matrix, Alicon PrioTrap®, which binds with high affinity and specificity to prion proteins. Thus we were able to identify prion protein (PrP^C^)–the precursor of prions (PrP^Sc^)–in milk from humans, cows, sheep, and goats. The absolute amount of PrP^C^ differs between the species (from µg/l range in sheep to ng/l range in human milk). PrP^C^ is also found in homogenised and pasteurised off-the-shelf milk, and even ultrahigh temperature treatment only partially diminishes endogenous PrP^C^ concentration.

**Conclusions/Significance:**

In view of a recent study showing evidence of prion replication occurring in the mammary gland of scrapie infected sheep suffering from mastitis, the appearance of PrP^C^ in milk implies the possibility that milk of TSE-infected animals serves as source for PrP^Sc^.

## Introduction

Prion protein was detected in attempts to identify the infective agent of TSE [1], [2]. The finding that prion protein is present in normal and TSE-infected brain at similar levels [3], [4] suggests that the “cellular” prion protein (PrP^C^) constitutes a precursor of the “scrapie” prion protein (PrP^Sc^) causing TSE such as bovine spongiform encephalopathy (BSE) in cattle or Creutzfeldt-Jakob disease (CJD) in humans. There is convincing evidence that the transition from precursor protein to infectious prion is due to a major conformational transition [5].

Prion protein is highly conserved among mammals [6]. It is primarily synthesized in cells of the central nervous system [7], but is also abundantly expressed in several peripheral tissues [8], [9]. An amino-terminal signal sequence targets prion protein to the endoplasmatic reticulum, where it transits the Golgi and ultimately reaches the external surface of the cell membrane [10]. There it is attached to a carboxy-terminal glycosyl phosphatidylinositol anchor [11]. The mature bovine protein of 217 amino acids contains two consensus acceptor sites for addition of N-linked polysaccharides [12].

Prion proteins (PrP^C^ and PrP^Sc^) have been detected in the cellular fraction of blood [13]–[17], but so far not in milk [18]–[21]. Considering that milk and milk products represent a major component of human nutrition it seems of particular importance to analyze milk for the presence of prion proteins. A first step in this direction is to determine the amount of PrP^C^ in milk of healthy animals. If milk contains a significant amount of PrP^C^, this could indicate that PrP^Sc^ might be present in so far undetectable amounts in milk of TSE infected animals. However, the high concentration of total protein (about 40 mg/ml) and the high amount of lipids (about 35 mg/ml) in the milk make prion protein analysis by common biochemical methods demanding. We have therefore developed an adsorption matrix, Alicon PrioTrap®, which binds with high affinity and specificity to prion proteins PrP^C^ and PrP^Sc^. The exceptional binding properties of Alicon PrioTrap® result from hydrophilic and hydrophobic surface clusters that recognize different prion protein epitopes, allowing quantitative enrichment of extreme low quantity of prion proteins in body fluids and in biological tissues.

## Results

**Figure 1:**
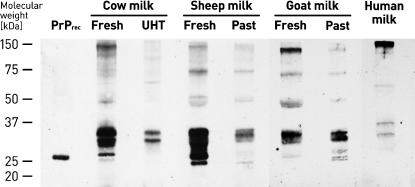
Detection of native PrP^C^ in milk of human and animals. A volume of 10 ml fresh, ultra-high temperature treated (UHT) and pasteurised (Past) milk were enriched for PrP^C^ using Alicon PrioTrap® technology. Concentrated PrP^C^ was analyzed by Western Blotting using PrP−mab 8B4 (Alicon AG). The molecular weight markers are indicated. Recombinant bovine PrP(25−241) (Alicon AG) was used as a standard.

The detection of native PrP^C^ after enrichment from 10 ml milk from cow, sheep, goat, and human using Alicon PrioTrap® is shown in figure 1. In cow milk three PrP^C^ isoforms are observed with an apparent molecular mass of about 34 kD, 30 kD, and 27 kD corresponding to diglycosylated, monoglycosylated, and unglycosylated PrP^C^, respectively. In some preparations monoglycosylated PrP^C^ appears as a double band, indicating that the two glycosylation sites may be linked to different carbohydrates. The apparent molecular mass of unglycosylated PrP^C^ is slightly higher when compared to a recombinant bovine PrP(25–241) standard at 26 kD, indicating that native PrP^C^ in milk contains a glycosyl phosphatidylinositol anchor [11]. About the same distribution of PrP^C^ isoforms is observed for sheep, goat, and human milk, although the total amount of native PrP^C^ significantly differs between the species. The relative ratio of sheep/cow/goat/human PrP^C^ is estimated at 100/20/4/1. From experiments performed on sequential incubations with Alicon PrioTrap® the total concentration of PrP^C^ in fresh cow milk can be estimated to be about 200 pg/ml. Taking into account the relative ratios of PrP^C^ in milk of different species, fresh sheep milk and goat milk contain about 1 ng/ml and 40 pg/ml PrP, respectively. Human breast milk contains less than 10 pg/ml PrP^C^. The concentration of PrP^C^ in Swiss off-the-shelf milk is reduced when compared to fresh milk, but prion protein can clearly be detected (Figure 1). About the same concentration of PrP^C^ was measured for organic farm milk and non-organic farm milk as well as for pasteurized and ultra-high temperature (UHT) treated milk (data not shown).

**Figure 2:**
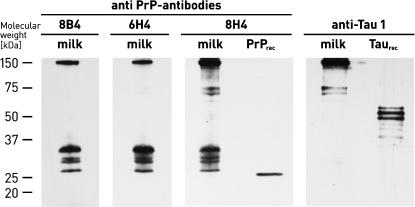
Specific binding of anti-PrP monoclonal antibodies to milk PrP^C^. Various anti-PrP monoclonal antibodies were used for detection of PrP^C^ in fresh cow milk. A Tau-1 protein-specific monoclonal antibody was used as a negative control. Recombinant bovine PrP(25−241) (Alicon AG) and recombinant Tau-1 protein (Chemicon International) were used as a standard.

To confirm specificity of immunochemical detection of PrP^C^ in milk, we compared different anti-PrP monoclonal antibodies, which are directed against non-overlapping epitopes (Figure 2): PrP−mab 8B4 binds to residues 37−44 of mouse PrP [22]; mAB 6H4 targets residues 144−152 [23]; and PrP−mab 8H4 binds to residues 175−185 [24]. The three antibodies recognize the same proteins and thus confirm the presence of PrP^C^ in milk. In control experiments, with non-PrP antibodies, *e.g.*, anti-Tau protein monoclonal antibody (Chemicon International) (Figure 2) and anti-Aβ monoclonal antibody (Calbiochem, Germany; data not shown), none of the PrP^C^ isoforms was detected, thus confirming binding specificity of the anti-PrP monoclonal antibodies. An interesting observation with regard to antibody 8B4 is its “clear” detection profile when compared to 6H4 and 8H4 antibodies. This can be rationalized by 8B4 not recognizing a variety of carboxy-terminal fragments of milk PrP^C^, which appear as smear in the Western Blot.

**Figure 3:**
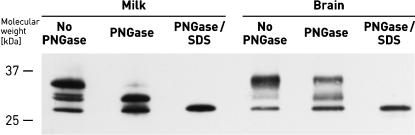
Glycoform patterns of milk and brain PrP^C^. Alicon PrioTrap®-enriched PrP^C^ from cow milk and brain homogenate was treated with PNGase or PNGase/SDS before Western Blotting using PrP−mab 8B4.

We further compared the glycoforms of native prion protein in cow milk with those of bovine brain, a tissue where prion protein expression is well characterized. The glycoforms were identified by digestion with PNGase (Figure 3), an enzyme that cuts off oligosaccharides from N-linked glycoproteins, *e.g.*, the two N-linked sugars of PrP^C^ [12]. After partial cleavage with PNGase the upper PrP-isoform in the Western Blot representing diglycolysated PrP^C^ (34 kD) disappears in favour of monoglycosylated (30 kD) and nonglycosylated PrP^C^ (27 kD). In parallel, there seems to be a small shift from the higher molecular weight monoglycosylated form to the lower molecular weight form. A slight downshift of the monoglycosylated PrP^C^ is also observed for brain homogenate after PNGase treatment (Figure 3). The diglycosylated PrP^C^ isoforms differ slightly in molecular mass, indicating that carbohydrate structure of PrP^C^ in milk and brain may not be identical. More stringent reaction conditions result in complete truncation of carbohydrates from PrP^C^. Most importantly, the apparent molecular masses of nonglycosylated PrP^C^ in milk exactly matches with that of the corresponding PrP^C^ in brain homogenate.

**Figure 4:**
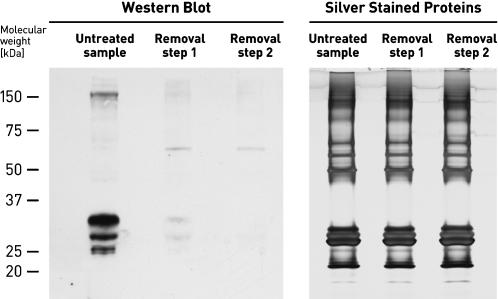
Elimination of PrP^C^ from milk. PrP^C^ from 10 ml fresh cow milk was detected as described in figure 1, followed by two consecutive PrP^C^ removal steps, where the milk supernatant was incubated with Alicon PrioTrap® filtration resin for 30 min before immunochemical detection. The total amount of milk protein was analyzed by Silver Stain analysis.

Alicon PrioTrap® can also be applied for elimination of prion protein from milk. As shown in Figure 4, after the first treatment of 10 ml milk with Alicon PrioTrap® more than 95% of endogenous PrP^C^ was already removed, and after the second treatment PrP^C^ was completely eliminated. However, the overall protein concentration (measured by bicinchoninic acid assay, Pierce) was constant with about 40 mg/ml before and after PrP^C^ elimination. The protein composition of milk as analyzed by SDS PAGE (Figure 4) was not affected either. Prion protein was also completely removed, when milk was spiked with PrP^Sc^ from mouse Rocky Mountain Laboratory (RML) brain homogenate (data not shown). Thus, Alicon PrioTrap® can be used for enrichment and detection of overall prion protein in milk, but also for complete removal of prions.

## Discussion

Milk contributes 13% to the worldwide protein supply for humans. World milk production ranges around 500 million tons a year. Before fresh milk reaches the consumer, it is usually homogenized to reduce fat particle size in order to increase digestibility of the milk and heated. Surprisingly, pasteurisation (heating for 30 seconds to 72°C) and ultra-high temperature treatment (heating for 1–4 seconds to 135°C) only leads to a partial reduction of the amount of PrP^C^. This supports the observation that PrP^C^ is highly stable in milk. Thus, the heating procedures used to inactivate DNA-containing pathogens are not sufficient to eliminate endogenous prion proteins.

The presence of PrP^C^ in blood has been documented [14], [15], and is confirmed by our own unpublished observations. To produce one liter of milk, about 400 to 500 liters of blood must pass through the udder of a cow. It is thus possible that the PrP^C^ found in milk derives from blood cells or, alternatively, has been secreted from glandular epithelial cells. Cell types that have been identified in milk from healthy cows are mainly macrophages, and other leucocytes. However, in our assay cells are completely removed by centrifugation. Therefore, the recovered PrP^C^ is not cell associated but most likely binds to other proteins or lipids resulting in stable molecular complexes. The fact that milk contains full-length PrP^C^, very likely comprising the glycolipid anchor, indicates that prion protein was originally cell-bound and does not represent any of the amino-terminal truncation products of PrP^C^ known to be released from normal cells under physiological conditions [25]. The detection of such a considerable expression of cell membrane bound or derived PrP^C^ in milk constitutes one of the key requirements for the generation of infectious prions in the udder of infected animals.

Over the last 10 years, scientific groups, risk assessment agencies, and public health organizations have debated the TSE risk for milk and milk products [26], [27]. Epidemiological and bioassay data so far available have not provided evidence for milk to harbour prion infectivity and infectious prions have as yet not been detected by bioassays in the milk, colostrum or udder of clinical BSE cases in cow [18]–[21]. However, a recent statement of the European Food Safety Authority affirmed that based on a number of observations from research data, there are indications that infectivity in the milk from small ruminants can not be totally excluded [28]. Furthermore, the exclusion of animals with mastitis, an inflammation of the mammal gland, being able to destabilize the blood-milk barrier, is considered a measure able to reduce but not to eliminate the potential contamination risk [28]. The rational of this conclusion is confirmed by a recent study showing that in sheep naturally affected with both scrapie and lymphocyte or lymphofollicular mastitis, PrP^Sc^ accumulation was present in lymphoid follicles adjacent to milk ducts [29]. At least in natural sheep scrapie, prion replication can occur following a lymphotropic virus infection in the inflamed mammary gland. This study has not detected PrP^Sc^ or prion infectivity in milk itself. However, since under such inflammatory conditions, the total number of immune cells increases in milk of animals, it might be possible that infectious PrP^Sc^ is also passing through and reaches the milk. In this context milk from such animals could possibly be responsible for the spread of scrapie from the ewes to their offspring in affected sheep or goat flocks. Moreover, sheep and goat milk could also constitute a TSE exposure risk for mammals (humans) consuming these products.

The former Scientific Steering committee of the European Commission and the European Food Safety Authority recommend that research should intensify on the safety of milk of small ruminants with regard to TSE risk. Limited new data are expected to be published in the near future and there is still little research initiated in this area [28]. The Alicon PrioTrap® technology opens a new avenue for studying the biochemical characteristics of prion protein in milk and thus may contribute to offer a feasible approach to perform an appropriate study on the milk safety with regard to TSE risk.

## Materials and Methods

### Preparation of milk samples

Fresh milk was obtained from healthy individuals and transported at 4°C. Cow UHT milk, sheep and goat pasteurised milk were obtained from the Swiss market. Each sample was prepared from 10 ml milk, centrifuged at 3000× g for 10 min to ensure complete removal of cells.

### Preparation of brain homogenate

10% (w/v) bovine brain homogenate was prepared in 100 mM Tris-HCl pH 7.5 containing 2% sodium lauryl sarcosinate. This solution was diluted in 100 mM sodium phosphate buffer pH 8 containing 0.5% NP-40 to obtain 1% (w/v) brain homogenate.

### Concentration of milk PrP^C^

The milk supernatant was stirred for 30 min in the presence of Alicon PrioTrap®. The resin was centrifuged for 2 min at 2000× g and washed three times at RT with 10 ml washing solution containing 100 mM sodium phosphate buffer pH 8.

### Immunochemical PrP^C^ detection

Concentrated milk and brain PrP^C^ was denaturated in SDS sample buffer and heated at 70°C for 10 min and at 95°C for 5 min, respectively. Samples were applied to a 12% SDS polyacrylamide gel for electrophoresis and subsequently transferred to a PVDF membrane. The membrane was blocked with 2% ECL Advance™ blocking agent (Amersham) if probed with 8B4 antibody or 1% bovine serum albumin if probed with other antibodies used in this study. 8B4 antibody was incubated at a concentration of 450 ng/ml, 8H4 at 85 ng/ml, 6H4 at 285 ng/ml, and anti-Tau-1 at 50 ng/ml. Sheep anti-mouse IgG horseradish peroxidase-conjugated secondary antibody (Amersham) was incubated at a 1/20,000 dilution. The immunoreactivity was visualized by chemiluminesecence detection following the manufacture's instruction (ECL Advance Western Blot detection Kit, Amersham).

### PNGase treatment of milk and brain PrP^C^

For PNGase treatment prion protein extracted from 10 ml cow milk or 10 µl of 1% (w/v) cow brain homogenate was incubated for 12 h at 37°C in buffer containing 100 mM sodium phosphate, 10 mM Tris-HCl, 1% NP-40, 1% MEGA-8, pH 8, and 1.5 units of N-Glycosidase F (Roche). Under more stringent cleavage conditions, proteins were denatured by heating for 10 minutes at 100°C in the presence of 0.5% SDS before treatment with 4 units of N-Glycosidase F.

### Total milk protein detection

A milk volume corresponding to 40 µg total protein (1 µl) was heated in SDS loading buffer at 70°C for 10 min. After electrophoresis on a 12% SDS polyacrylamide gel, the proteins were detected by silver staining (SilverSNAP Stain kit II, Pierce).
